# Health Risk Assessment of Heavy Metals from Smoked *Corbicula fluminea* Collected on Roadside Vendors at Kelantan, Malaysia

**DOI:** 10.1155/2019/9596810

**Published:** 2019-09-30

**Authors:** Koh Han Dee, Faizuan Abdullah, Siti Nor Aini Md Nasir, Suganthi Appalasamy, Rozidaini Mohd Ghazi, Aweng Eh Rak

**Affiliations:** ^1^Department of Natural Resource and Sustainability, Universiti Malaysia Kelantan, Jeli Campus, Locked Bag No. 100, 17600 Jeli, Kelantan, Malaysia; ^2^Department of Chemistry, Faculty of Science, Universiti Teknologi Malaysia, 81310 UTM Johor Bahru, Johor, Malaysia

## Abstract

*Corbicula fluminea* serves as traditional food to the local people in Kelantan, Malaysia. Concerns regarding river contamination, smoking method, and associated adverse effects on public health had been increasing. Hence, this study aims to measure the level of heavy metals (Cd, Cu, Mn, Pb, and Zn) and assess human health risk in *C. fluminea* consumption at Kelantan. Heavy-metal analysis was done using flame atomic absorption spectrophotometry, while human health risk was assessed using provisional tolerable weekly intake (PTWI), target hazard quotient (THQ), and hazard index (HI). The estimated weekly intake (EWI) for all metals was found within PTWI, while THQ for Cd, Cu, Mn, Pb, and Zn was 0.12, 0.06, 0.04, 0.41, and 0.03, respectively. The HI was calculated at 0.61 which is less than 1, considered as the safe consumption level. Therefore, *C. fluminea* consumption in this study was found safe from the health risk of noncarcinogenic effect over a lifetime.

## 1. Introduction

*Corbicula fluminea* (Müller, 1774) is known as “Etak” at Kelantan, Malaysia. The local people consume *C. fluminea* since a very long time ago, especially smoked *C. fluminea* as their favourite. Smoked *C. fluminea* is broadly sold at morning market, night market, and in the street stalls. This food generates income for local Kelantanese. In average, *C. fluminea* sellers can generate up to 600 USD/month [1]. Different countries have different ways to cook *C. fluminea*. This can be shown in the Philippines where *C. fluminea* is consumed raw-pickled or cooked in soup [2]. In Kelantan, the local people consume *C. fluminea* in many cooking methods, including the smoke method, sun-drying method, and frying method [1]. Among these cooking methods, the smoke method is the most popular method. The local people consume the smoked *C. fluminea* while they are watching television, studying, and chatting with friends.

*Corbicula fluminea* is distributed in most of the freshwater river in Malaysia, such as Perak River, Pahang River, Golok River, and Pergau River. This clam is mostly found in sand and gravel sediments. *C. fluminea* obtains the food in water column and sediment via inherent filter feeding and pedal feeding ability, respectively [3]. They mainly feed on suspended matter, phytoplankton, bacterioplankton, zooplankton, diatoms, green algae, and protozoans [4, 5]. With this feeding ability, *C. fluminea* is important in ecosystem balance by contributing in benthic/pelagic biogeochemical coupling and enhanced oxygen penetration into the sediment [6, 7]. *C. fluminea* is also used as a bioindicator of the heavy metals and microplastic in freshwater river [8, 9].

In Kelantan, high demand for *C. fluminea* is contributed by a better understanding of nutrition content on seafood consumption. *C. fluminea* is well known for its high nutritional values of protein, omega-3 fatty acid, and pharmaceutical values including lowering cholesterol accumulation, increasing antioxidant, and anticancer and antitumor activities [10–13]. Despite the ecological importance and economic benefits, the heavy metals potentially accumulate in *C. fluminea* soft tissue [5]. The river pollution and popular smoking method are suspected to contaminate *C. fluminea*. One of the most common heavy metals bioaccumulation in human body is through the food chain and regular consumption of contaminated food [14]. The relationship between the heavy metals concentrations in this clam and associated health risk assessment in Kelantan is yet to e reported. The local people lack information about the safety of *C. fluminea* consumption. Hence, this research aims to determine the selected heavy-metal (cadmium, copper, manganese, lead, and zinc) level in *C. fluminea* soft tissue that is sold in the streets and assess the human health risk. These selected heavy metals and their effects on human health are of great concern based on the previous studies [5, 15–17]. The health risk assessment is carried out on the ready-to-eat *C. fluminea* sold along the roadside. The provisional tolerable weekly intake (PTWI), target hazard quotient (THQ), and hazard index (HI) were used to evaluate the health risk.

## 2. Materials and Methods

### 2.1. Study Area

**Figure 1:**
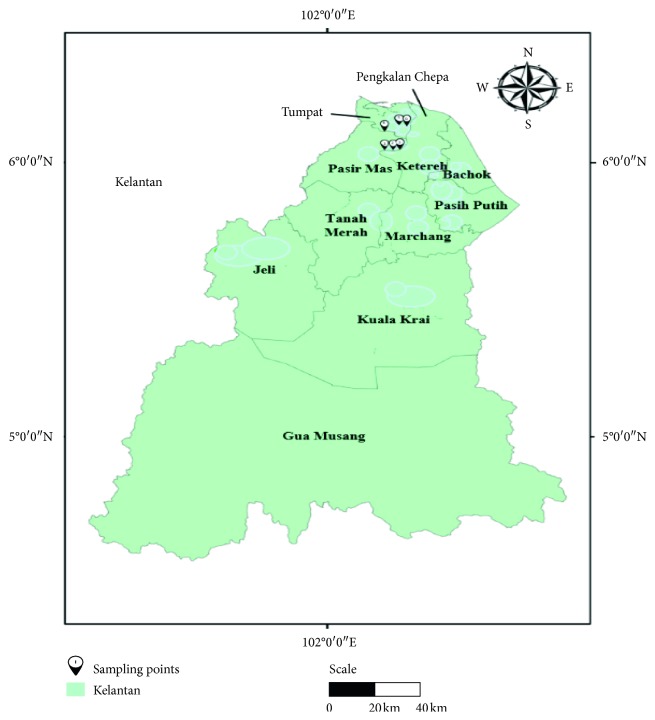
The map of the Kelantan state, Malaysia. The study area and sampling points of *C. fluminea* were at Pasir Mas and Tumpat, Kelantan.

The samples were collected at Pasir Mas and Tumpat, Kelantan (6.0495938 N, 102.1693547 E and 6.1467273 N, 102.218605 E), for six months duration. Six stalls of *C. fluminea* were selected due to their availability throughout the year (Figure 1). The primary source of *C. fluminea* used in these stalls is from Perak River which is located close to oil palm plantations, durian farms, and urban area. Around 1 kg smoked *C. fluminea* was collected from each seller for the heavy-metal analysis. Each sample was labelled with the date, time, collection site, and weather. The samples were preserved at 4°C by storing in an ice box during their transportation to the laboratory [18]. The samples were stored in a freezer at −20°C before analysis.

### 2.2. Sample Analysis

The soft tissues of *C. fluminea* were obtained using a blade and oven dried (60°C, 72 hours) to constant weight. After that, the samples were ground into homogenous samples using a mill machine (Panasonic®MX-900M). The samples were preserved in amber jars and stored in a desiccator before acid digestion. The digestion process was carried out by following the standard method by PerkinElmer Corporation [19].

Approximately 5 g of dried samples was placed in the 250 mL beaker in which 5 mL of 95% sulfuric acid (Merck) and 5 mL of 65% nitric acid (Merck) were added. The samples were covered immediately with watch glass. After the reaction between sample and acid was completed, the mixtures were heated at 60°C for 30 minutes using hot plates (IKA® C-MAG HS 7). The samples were allowed to cool, and 20 mL of 65% nitric acid was added. The temperature was increased slowly to 150°C for two hours. Then, the solutions were allowed to cool, and 1 mL of 30% hydrogen peroxide (Merck) was added until the colour became clear. Samples were diluted to 50 ml with deionized water. The solutions were then filtered by filter paper (Whatman® No. 41 filter circles, 125 mm) and syringe filter (Minisart® nylon syringe filter, 25 mm).

The samples analysis was analysed by using the flame atomic absorption spectrophotometer PinAACLE 900F and measured in triplicates. The accuracy and validity of the applied protocol were evaluated using the Standard Reference Materials (SRM 2976, National Institute of Standards and Technology) due to its suitability in the mussel sample and matrix. Recovery rates were found to be between 99.98%–110.08% for the selected metals.

### 2.3. Human Health Risk Assessment (HHRA)

All data obtained were converted into wet weight (ww) using a conversion factor of 0.19. The conversion factor is based on the moisture analysis carried out in the food laboratory, Universiti Malaysia Kelantan by our research assistant, Siti Nor Aini Md Nasir, on 28 October 2018.

(a) Maximum permissible limit (MPL) of the heavy metals for the consumers set by the European Union [20] and FAO compilation of the legal limits by FAO [21].
(b) The estimated daily intakes (EDIs) of the consumers in order to evaluate the adverse health effects:
The JECFA uses the term estimated daily intake (EDI) and provisional tolerable daily intake (PTDI) for contaminants as heavy metals that can accumulate in the human body [22]. Estimated daily intake (EDI) (*μ*g/kg body weight) of heavy metals from *C. fluminea* consumption was obtained using the following equation (1) [23, 24]:
  (1) $$\begin{array}{c} \text{EDI}=\frac{\left(C_{\text{metal}}\times \text{IR}\right)}{\text{BW}}, \end{array}$$
where EDI is estimated daily intake and *C*_metal_ (mg/kg ww) is an average weighted heavy metal content in *C. fluminea.* Ingestion rate (IR) (gram/day person) is the daily mussel consumption. BW is the average body weight. The average IR is determined by interviewing the local people using a simple questionnaire. The average IR of *C. fluminea* for Malaysian adults is 75 g/day person which is similar to the literature [2]. The average body weight for Malaysians was 62.65 kg [25].
To obtain estimated weekly intake (EWI), the EDI will be multiplied by a factor of 7 corresponding to 7 days. However, based on questionnaires and interviews, the local people consume *C. fluminea* with a frequency of three days per week. Hence, EWI was obtained using the following equation:
  (2) $$\begin{array}{c} {\text{EWI}}_{\text{this study}}=\text{EDI }\times 7\times \frac{3}{7}. \end{array}$$
The EWI_this study_ is compared to the JECFA provisional tolerable weekly intake (PTWI).
(c) Target hazard quotient has been recognised as a useful parameter for evaluation of risk associated with the consumption of metal contaminated food. To assess the noncarcinogenic risk due to heavy metal exposure in *C. fluminea* soft tissue, the target hazard quotient (THQ) was computed using the following equation [26]:
  (3) $$\begin{array}{c} \text{THQ}=\left[\frac{{\text{EF}}_{r}\times \text{ED}\times \text{IR}\times \text{MC}}{\text{RfD}\times \text{BW}\times \text{AT}}\right]\times 10^{-3}, \end{array}$$
where EF_*r*_ is exposure frequency (156 days per year for the average consumer, according to the local interview); ED is exposure duration (70 years) equivalent to the average human lifespan; IR is the ingestion rate (75 grams per person per day); MC is the metal concentration in *C. fluminea* soft tissue (in mg/kg, ww); RfD is the oral reference dose (Cd is 1 mg/kg wt/day, Cu 40 mg/kg wt/day, Zn 300 mg/kg wt/day, and Mn 140 mg/kg wt/day) [27]. The reference dose (RfD) is an estimate of the daily intake of contaminant during a lifetime that would not cause adverse health effects to the human [28]; BW is the average body weight (62.65 kg); AT is the averaging time for noncarcinogens (i.e., 30 years or 10,950 days).
With the refusal to set the reference dose of Pb by EPA [29], the THQ for Pb was calculated by using the following equation [30]:
  (4) $$\begin{array}{c} \text{THQ}=\frac{{\text{C}}_{\text{Pb}}}{\text{MPL}}, \end{array}$$
where C_Pb_ is the Pb concentration in *C. fluminea* (mg/kg ww). MPL: maximum permissible limit. In this study, the MPL of Pb for bivalve is 1.50 mg/kg ww [20].
THQ < 1 indicates no obvious risk. A THQ >1 shows that the level of exposure is higher than the oral reference dose (ORD), which assumes that a daily exposure at this level is likely to cause harmful health effects during the lifetime in a human population [31]. Following EPA guidelines, we assumed that the ingested dose was equal to the absorbed contaminant dose.
(d) The hazard index (HI) calculated from THQ is the total of the hazard quotients [23, 32]:
  (5) $$\begin{array}{c} \text{HI}={\text{THQ}}_{\text{Cd}}+{\text{THQ}}_{\text{Cu}}+{\text{THQ}}_{\text{Mn}}+{\text{THQ}}_{\text{Pb}}+{\text{THQ}}_{\text{Zn}} \end{array}$$
If HI less than 1, there is no obvious risk.

### 2.4. Statistical Analysis

The metal concentrations for six sellers were averaged to obtain the mean. Microsoft Excel was used to tabulate all the data throughout the experiment and generate the hazard index graph. SPSS 23 for Windows was used to run the statistical analysis including the mean, standard error, and one-way ANOVA with Tukey post hoc test. The statistical significance was significant when *P* < 0.05.

## 3. Results and Discussions

### 3.1. Heavy-Metal Concentration in *C. fluminea* and Human Health Risk Assessment

The mean concentration of Cd, Cr, Cu, Mn, Pb, and Zn (mg/kg ww) in *C. fluminea* from the roadside stalls, which are consumed by local people, is tabulated in Table 1. The values in each month are the mean from six stalls. Metal concentration in *C. fluminea* exhibited an order of Zn > Mn > Cu > Pb > Cd.

In this study, dietary exposure to heavy metals through consumption of *C. fluminea* in the studied areas was evaluated using estimated daily intake (EDI) by considering the average concentration of the heavy metals and the respective consumption rate for adults. Then, the estimated weekly intake (EWI) was calculated and compared with PTWI.  Table 2 shows the values of EDI, EWI, PTWI, and EWI :PTWI ratio.

### 3.2. Cadmium

Cd detected in *C. fluminea* ranged from 0.17 to 0.34 mg/kg ww and has a mean of 0.23 ± 0.01 mg/kg ww. No significant difference was found between Cd levels in each month with *P* > 0.05. The Cd level is within the permissible limit set by the European Union of 1.0 mg/kg ww [20]. In EWI measurement, the local people consuming *C. fluminea* would intake 0.84 *μ*g/kg/week or 0.05 mg/62.65 kg person/week (0.84 × 62.65/1000) of the Cd. The EWI : PTWI ratio of the Cd was 12.00%. This showed that the intake is within the safe level.

The current result is found to be higher than that in the study by Zhelyazkov et al. [35] who reported the EWI of Cd for the marine mollusc (*Mytilus galloprovincialis*) was 0.002 mg/70 kg person/week (0.0032 × 70 × 7/1000). Besides, Yunus et al. [36] study the metal level in the cockles (*Anadara granosa*) from Kuala Selangor, Malaysia. They reported 5.96 *μ*g/70 kg/daily or 0.04 mg/70 kg person/week (5.96 × 7/1000) of Cd intake with cockle consumption which is also lower than the EWI value of Cd in the current study. Sharif et al. [37] study on consumption of shellfish clam (*Metretrix* spp.), scallop (*Amusium pleuronectes*), and conch (*Strombus canarium*) at Kudat, Sabah. They reported that if the local people consume these shellfish every day, the EWI values of Cd for *Metretrix* spp., *Amusium pleuronectes*, and *Strombus canarium* were 0.01 mg/62.65 kg person/week (0.03 × 7 × 62.65/1000), 0.69 mg/62.65 kg person/week (1.58 × 7 × 62.65/1000), and 0.009 mg/62.65 kg person/week (0.02 × 7 × 62.65/1000), respectively.

EWI of Cd (0.05 mg/62.65 kg person/week) in the current study is higher than that of previous studies, except the scallop (*Amusium pleuronectes*). High EWI of Cd in *C. fluminea* is due to high ingestion rate and high Cd concentration in *C. fluminea*. Cd is mainly used in industries includes electroplating, alloy production, pigments, and battery production [38]. This is in agreement in the current study where metal industries, automobile workshops, and laptop shops can be found near to Perak River. The Cd in discharged wastewater is easily deposited into river, transferred to aquatic life, and eventually accumulated in human body through the food chain [39]. As a filter feeding organism, *C. fluminea* is reported susceptible to heavy-metal exposure in river and possess high metal level [5].

Cadmium is a nonessential element for human and detrimental to our health even when ingested in small quantities. Food is the primary source of Cd exposure, besides cigarette smoking [40, 41]. Continuous Cd consumption leads to respiratory system damage, lung cancer, Parkinson's and Wilson's diseases, and estrogen receptor-positive breast cancer in postmenopausal women [42, 43]. In Japan, consumption of Cd-contaminated rice causes osteomalacia (bone disease) and kidney malfunction to the local people [44]. Cd-poisoning patients need to be treated with gastrointestinal tract irrigation, supportive care, and chemical decontamination with chelating agents and nanoparticle-based antidotes [45].

### 3.3. Copper

The Cu detected in *C. fluminea* ranged from 2.64 to 12.61 mg/kg ww and has a mean of 5.03 ± 0.47 mg/kg ww. There is no significant difference between the Cu level in every month with *P* > 0.05, except in April with *P* < 0.05. The Cu level is within the permissible limit of 20 to 70 mg/kg ww set by FAO compilation of the legal limits [21]. In EWI measurement, the *C. fluminea* consumer would intake 18.06 *μ*g/kg/week or around 1.13 mg/62.65 kg person/week (18.06 × 62.65/1000) of the copper. The EWI : PTWI ratio of the Cu was 0.52%. This showed that the intake is within the safe level. The result obtained is higher than the previous study carried out by Olmedo et al. [46] who reported 0.82 mg/60 kg person/week (0.117 × 7) of Cu intake through fish and shellfish consumption in Andalusia (Southern Spain). Bat et al. [47] study the heavy metals in *Mytillus galloprovincialis* from the Turkish Black Sea coasts and found that EWI of Cu was in the range of 0.24 to 0.88 mg/70 kg person/week (0.0035 × 70 to 0.0126 × 70) with EWI :PTWI ratio 0.1% to 0.36%.

By comparing the data with the mussel in the previous studies, it is notable that the Cu level in the current study was higher than that of those studies. It is believed that using copper-based pesticide in paddy fields in Perak River and discharge of municipal sewage increase the Cu concentration in the soil [48, 49]. The contaminant leached into the river and the sediment eventually accumulated in *C. fluminea* soft tissue [50]. This is supported by Patrick et al. [6] who reported *C. fluminea* soft tissue had significantly greater Cu concentrations compared to river and sediment.

Copper is an essential trace element for living organisms which allows the critical enzyme to function properly and assists enzyme in transferring energy into the cells in humans [51]. The shellfish is an excellent source of Cu for human needs [46]. However, higher copper uptake than needed is a double-edged sword that causes adverse effects [52]. The effects include headache, vomiting, liver and kidney damage, and Wilson's disease [53].

### 3.4. Manganese

The Mn level in *C. fluminea* ranged from 3.63 to 26.16 mg/kg ww and has a mean of 10.25 ± 1.92 mg/kg ww. No significant difference was found between Mn level in each month with *P* > 0.05. There is no permissible limit for manganese. In EWI measurement, the *C. fluminea* consumers would intake 36.81 *μ*g/kg/week or around 2.31 mg/62.65 kg person/week (36.81 × 62.65/1000) of the manganese. The EWI : PTWI ratio of the Mn was 3.76%. This showed that Mn intake is within the safe level. The current result is found to be higher than that of the study by Olmedo et al. [46], who reported 0.35 mg/60 kg person/week (0.05 × 7) of Mn intake through fish and shellfish consumption in Andalusia. The current result is also found to be slightly higher than that of the study by Bat et al. [47] who reported that EWI of the Mn in *M. galloprovincialis* was in the range of 1.37 to 2.21 mg/70 kg person/week (0.0196 × 70 to 0.0315 × 70).

High Mn contamination in *C. fluminea* is associated with accumulation from the sediment and river. This is supported by Hulten et al. [54] who reported that sediment is the main source of Mn, specifically on the surface of the sediment particles. Moreover, wastewater discharged from the metal industry flows into the river, lowering its pH and facilitating the sediment dissolution into the river [55]. Hence, concentrated Mn in river and sediment contributes to high Mn level in *C. fluminea*.

Manganese is an essential element for living organisms. Tiny amounts of Mn are required to form healthy bones, regulate the blood sugar level, maintain the metabolism, promote the digestion, and boost the vitamin absorption [56]. Overconsumption of manganese from food sources is rare [57]. When Mn uptake exceeded the required amount, it has negative impacts on the human body including weakness, muscle pain, less facial expression, and clumsy movement of the limbs and neurological damage [58, 59].

### 3.5. Lead

Pb detected in *C. fluminea* ranged from 0.17 to 0.34 mg/kg ww and has a mean of 0.62 ± 0.12 mg/kg ww. The Pb detection in April and June is significantly different than that in other months with *P* < 0.05. The Pb level is within the permissible limit set by the European Union of 1.5 mg/kg ww [20]. In EWI measurement, the local people consuming *C. fluminea* would intake 2.22 *μ*g/kg/week or around 0.14 mg/62.65 kg person/week (2.22 × 62.65/1000) of the lead. The EWI :PTWI ratio of Pb was 8.88%. This showed that the intake rate of Pb is within the safe level.

The current finding is compared with that of the previous studies. Zhelyazkov et al. [35] reported that the EWI of Pb for the *Mytilus galloprovincialis* was 0.001 mg/70 kg person/week (0.0028 × 70 × 7/1000). Besides, Sharif et al. [37] reported that the EWI of Pb for *Metretrix* spp., *Amusium pleuronectes*, and *Strombus canarium* were 0.04 mg/62.65 kg person/week (0.10 × 7 × 62.65/1000), 0.11 mg/62.65 kg person/week (0.25 × 7 × 62.65/1000), and 0.08 mg/62.65 kg person/week (0.19 × 7 × 62.65/1000), respectively. Bat et al. [47] reported that the EWI of Pb in *M. galloprovincialis* from the Turkish Black Sea coasts was in the range of 0.03 to 0.15 mg/70 kg person/week (0.00042 × 70 to 0.00217 × 70) with the highest EWI : PTWI ratio of 1.68 to 8.68%. In the current study, the Pb intake in weekly measurement is found to be higher than that of most of these previous studies. However, the current result is in line with a similar study by De la Cruz et al. [2] who also studied the human health risk in *C. fluminea* consumption in Philippines. They reported that EWI for average consumers was within 0.76 to 1.2 *μ*g/kg/day or 0.15 to 0.23 mg/65 kg person/week (0.76 × 65 × 3/1000 to 1.2 × 65 × 3/1000) assuming three times consumption per week as indicated in their result.

High Pb contamination in *C. fluminea* is due to fact that their habitat was within the vicinity of oil palm plantation that used pesticides and herbicides [60]. In Nigeria, Osobamiro and Adewuyi [61] reported that Pb concentration in oil palm plantation soil ranged from 15.5 to 33.1 mg/kg which is significantly higher than that of arable soil. The Pb-contaminated soils leached into the river and accumulated in the soft tissue of *C. fluminea* [62]. Besides, the smoking process of *C. fluminea* using firewood was also reported to increase Pb contamination in soft tissue of *C. fluminea* [63].

In the top 20 most poisonous heavy metals, Pb is the second element after As. Pb has no biological function in human body, and it is highly harmful to human health even in the smallest amount [64, 65]. There are many types of Pb exposures, but inhalation and ingestion through contaminated food is found to be the most common route [66]. Excessive Pb uptake causes intellectual damage to children such as cognitive and behavioural problems [67, 68]. This is because the brain development and central nervous system of children are susceptible to damage [69]. Long-term exposure to Pb-contaminated food caused the metal to be deposited into bone, leading to kidney and liver disease, cardiovascular disease, cancer, and reproductive system defect [64, 70].

### 3.6. Zinc

The Zn concentrations in *C. fluminea* were found in the range of 2.47 to 33.76 mg/kg ww with a mean of 17.49 ± 1.63 mg/kg ww. The Zn level in April is significantly higher than those in other months with *P* < 0.05. The Zn level is within the permissible limit in FAO compilation of the legal limits of 40–150 mg/kg ww [20]. This indicated that the Zn is in the safe level to consume. In EWI measurement, the *C. fluminea* consumers would intake 62.73 *μ*g/kg/week or around 3.93 mg/62.65 kg person/week (62.73 × 62.65/1000) of the zinc. The EWI : PTWI ratio of the Zn was 0.90%. The Zn intake is within the safe level. This result is in agreement with that of Olmedo et al.'s [46] study which found 3.25 mg/60 kg person/week (0.464 × 7) of Zn intake through fish and shellfish consumption in Andalusia (Southern Spain). The current result is lower than that in [47] which reported that the EWI of the Pb in *M. galloprovincialis* ranged from 3.92 to 13.23 mg/70 kg person/week (0.056 × 70 to 0.189 × 70) with the EWI : PTWI ratio of 0.86 to 2.70%.

The Zn in *C. fluminea* is believed to be due to phosphate rock fertilizer that is used for oil palm growth. Zn in the topsoil leached into the river [71]. Besides, Zn as a famous anticorrosive agent is widely used as antifouling paint for commercial boats [72, 73]. The leaching of boat paint coupling with accidental spill of oils or fuel increases the Zn contamination that accumulates in *C. fluminea* [74].

Zn is considered as an essential trace element for living organisms which is relatively less toxic compared to other metals. It assists in metabolism, enzyme catalytic activity, and immune system functioning and possesses antioxidant properties [75]. Oyster is reported as a source of Zn, followed by meat and mussel [76]. Excess Zn uptake damages the brain, respiratory tract, gastrointestinal tract, and prostate gland [77]. Besides, high Zn intake disrupts homeostasis for other essential elements and suppresses the Cu and Fe absorption [78].

**Figure 2:**
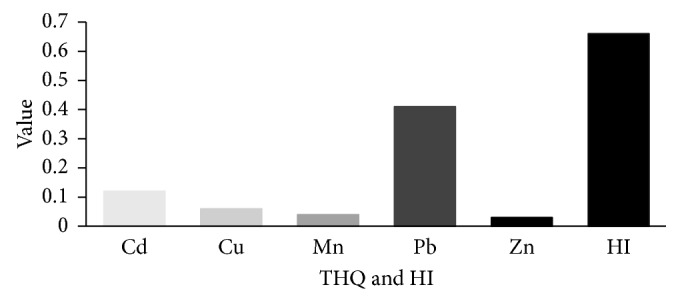
The THQ and HI values in this study. The THQ for each heavy metals and their hazard index (HI) were calculated from consumption of *C. fluminea* soft tissue collected from Pasir Mas and Tumpat, Kelantan.

Figure 2 shows the THQ values of the heavy metals and their hazard index (HI) for the consumption of the *C. fluminea*. The target hazard quotient (THQ) values of Cd, Cu, Zn, Pb, and Mn were 0.12, 0.06, 0.04, 0.41, and 0.03, respectively. The results of the THQ showed the following order of level: Pb > Cd > Cr > Cu > Zn > Mn. As presented in THQ, there exists no hazard. The HI index is found to be 0.61, which is less than 1. This suggests a relative absence of human health risk associated with intake of heavy metal via consumption of soft tissue alone. However, health risks to the consumers depend on the amounts of estimated weekly intake of heavy metal. In this study, we do not take into account the heavy-metal intake via other sources that probably contributes to higher THQ and HI. Hence, the present results need more attention in view of the fact that metals enter into the human body through other sources, primarily through other foodstuff.

The results in current study can be used as a guideline to consume *C. fluminea* safely. Nevertheless, as reported in the literature, the heavy metals in surrounding sediment and river water from anthropogenic activities correlate with heavy metal levels in *C. fluminea* [79, 80]. Although the level of heavy metal in *C. fluminea* is within the permissible limit, potential dangers emerging in the future depends upon industrial wastewaters and domestic activities. Given the vital level of *C. fluminea* to the local people, the routine biomonitoring of the clam for consumption should be done to ensure continuous food safety. The authority must ensure that domestic sewage and industrial effluents are treated before discharge into the rivers [81].

## 4. Conclusion

The mean concentrations of metals found in *C. fluminea* were 0.23 ± 0.01, 5.03 ± 0.47, 10.25 ± 1.92, 0.62 ± 0.12, and 17.49 ± 1.63 mg/kg ww for Cd, Cu, Mn, Pb, and Zn, respectively. All EWI values were found to be less than the JECFA provisional tolerable weekly intake (PTWI) value for studied heavy metals. Thus, there is no potential health risk for people who consume *C. fluminea* in Kelantan. The present study showed that the consumption of *C. fluminea* from Pasir Mas and Tumpat, Kelantan, at the rate of 75 g/day/person with the frequency of three times per week most probably does not pose a health hazard of cancer to the local population.

## Figures and Tables

**Table 1:** Concentration of heavy metals (mg/kg ww) in *C. fluminea* soft tissue from February to July.

Month	Cd	Cu	Mn	Pb	Zn
	Mean ± SE	Mean ± SE	Mean ± SE	Mean ± SE	Mean ± SE
February	0.24 ± 0.02^a^	3.68 ± 0.22^a^	7.62 ± 3.09^a^	0.45 ± 0.21^a^	9.06 ± 2.19^a^
March	0.21 ± 0.01^a^	3.26 ± 0.18^a^	12.18 ± 1.44^a^	0.44 ± 0.02^ab^	15.03 ± 0.63^ab^
April	0.23 ± 0.01^a^	8.97 ± 1.22^b^	6.91 ± 0.94^a^	0.86 ± 0.04^b^	29.82 ± 1.89^c^
May	0.20 ± 0.01^a^	4.94 ± 0.64^a^	8.16 ± 1.49^a^	0.49 ± 0.05^ab^	18.31 ± 2.08^b^
June	0.24 ± 0.02^a^	4.23 ± 0.43^a^	12.52 ± 2.83^a^	0.88 ± 0.06^b^	16.67 ± 1.45^b^
July	0.26 ± 0.01^a^	5.12 ± 0.15^a^	14.11 ± 1.70^a^	0.57 ± 0.35^ab^	16.06 ± 1.55^ab^
Average	0.23 ± 0.01	5.03 ± 0.47	10.25 ± 1.92	0.62 ± 0.12	17.49 ± 1.63

Letters show significant differences among the months at *P* < 0.05. The data were presented as mean ± standard error.

**Table 2:** The comparison of the estimated daily intake and estimated weekly intake with the recommended values for *C. fluminea* consumption.

	*C. fluminea* (mg/kg ww)	EDI (*μ*g/kg/day)	ADI^a^ (*μ*g/kg/day)	EWI^b^ (*μ*g/kg/week)	PTWI (*μ*g/kg/week)	EWI : PTWI ratio (%)
Cd	0.23	0.28	1	0.84	7^c^	12.00
Cu	5.03	6.02	350	18.06	3500^c^	0.52
Mn	10.25	12.27	140	36.81	980^d^	3.76
Pb	0.62	0.74	3.57	2.22	25^c^	8.88
Zn	17.47	20.91	1000	62.73	7000^c^	0.90

^a^Accepted daily intake which was calculated from PTWI; ^b^estimated weekly intake with a frequency of three times consumption per week; ^c^PTWI set by FAO/WHO [33]; ^d^reference dose of Mn established by USEPA [34].

## Data Availability

The data of the heavy metals used to support the findings of this study are included within the article.

## Additional Points

Human health risk as a result of heavy metals intake due to *C. fluminea* consumption was evaluated. All metal concentrations in *C. fluminea* were within the permissible limits. Smoked *C. fluminea* in Kelantan is safe for human health with the current consumption rate.

## Supplementary Materials

Supplementary Materials Graphical overview starting from *C. fluminea* selling to the consumption safety.
