# Supplementary figures and images for: Health Risk Assessment of Heavy Metals from Smoked Corbicula fluminea Collected on Roadside Vendors at Kelantan, Malaysia
Source: Biomed Res Int. 2019 Sep 30;2019:9596810. doi: 10.1155/2019/9596810 (PMC6791190; doi:10.1155/2019/9596810)

15    **Graphical Abstract**

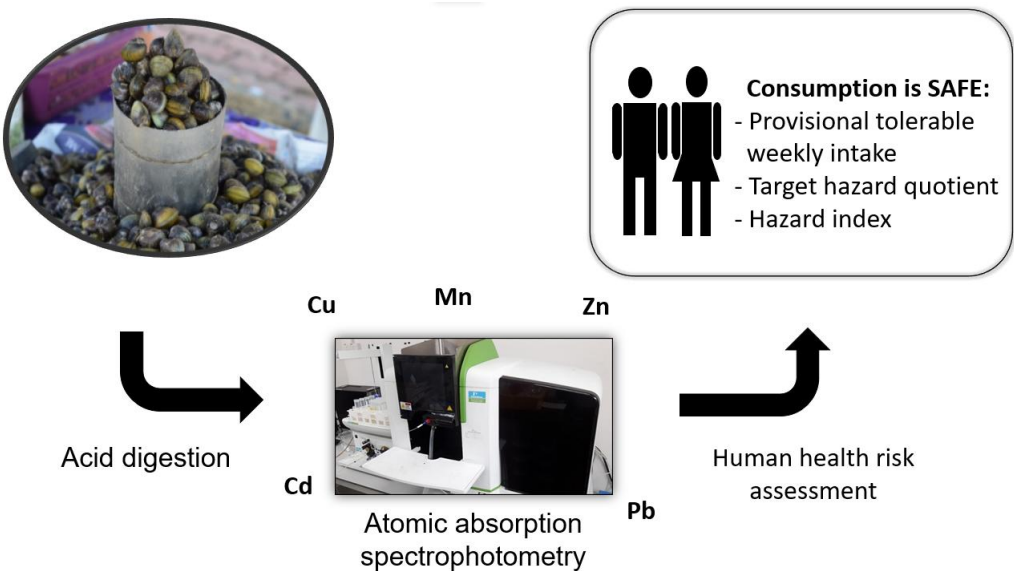

16  
17  
18

Supplement: Supplementary Materials — Graphical overview starting from C. fluminea selling to the consumption safety. [file 9596810.f1.pdf]
